# Analyzing Online Search Trends for Kidney, Prostate, and Bladder Cancers in China: Infodemiology Study Using Baidu Search Data (2011-2023)

**DOI:** 10.2196/57414

**Published:** 2025-03-14

**Authors:** Shuangquan Lin, Lingxing Duan, Xiangda Xu, Haichao Cao, Xiongbing Lu, Xi Wen, Shanzun Wei

**Affiliations:** 1 Urology Department The Second Affiliated Hospital of Nanchang University Nanchang University Nanchang China; 2 Urology Laboratory The Second Affiliated Hospital of Nanchang University Nanchang University Nanchang China

**Keywords:** bladder cancer, kidney cancer, prostate cancer, Baidu Index, infodemiology, public interest, patients’ concern

## Abstract

**Background:**

Cancers of the bladder, kidney, and prostate are the 3 major genitourinary cancers that significantly contribute to the global burden of disease (GBD) and continue to show increasing rates of morbidity and mortality worldwide. In mainland China, understanding the cancer burden on patients and their families is crucial; however, public awareness and concerns about these cancers, particularly from the patient’s perspective, remain predominantly focused on financial costs. A more comprehensive exploration of their needs and concerns has yet to be fully addressed.

**Objective:**

This study aims to analyze trends in online searches and user information–seeking behaviors related to bladder, kidney, and prostate cancers—encompassing descriptive terms (eg, “bladder cancer,” “kidney cancer,” “prostate cancer”) as well as related synonyms and variations—on both national and regional scales. This study leverages data from mainland China’s leading search engine to explore the implications of these search patterns for addressing user needs and improving health management.

**Methods:**

The study analyzed Baidu Index search trends for bladder, kidney, and prostate cancers (from January 2011 to August 2023) at national and provincial levels. Search volume data were analyzed using the joinpoint regression model to calculate annual percentage changes (APCs) and average APCs (AAPCs), identifying shifts in public interest. User demand was assessed by categorizing the top 10 related terms weekly into 13 predefined topics, including diagnosis, treatment, and traditional Chinese medicine. Data visualization and statistical analyses were performed using Prism 9. Results revealed keyword trends, demographic distributions, and public information needs, offering insights into health communication and management strategies based on online information-seeking behavior.

**Results:**

Three cancer topics were analyzed using 39 search keywords, yielding a total Baidu Search Index (BSI) of 43,643,453. From 2011 to 2015, the overall APC was 15.2% (*P*<.05), followed by –2.8% from 2015 to 2021, and 8.9% from 2021 to 2023, with an AAPC of 4.9%. Bladder, kidney, and prostate cancers exhibited AAPCs of 2.8%, 3.9%, and 6.8%, respectively (*P*<.05). The age distribution of individuals searching for these cancer topics varied across the topics. Geographically, searches for cancer were predominantly conducted by people from East China, who accounted for approximately 30% of each cancer search query. Regarding user demand, the total BSI for relevant user demand terms from August 2022 to August 2023 was 676,526,998 out of 2,570,697,380 (15.74%), representing only a limited total cancer-related search volume.

**Conclusions:**

Online searches and inquiries related to genitourinary cancers are on the rise. The depth of users’ information demands appears to be influenced by regional economic levels. Cancer treatment decision-making may often involve a family-centered approach. Insights from internet search data can help medical professionals better understand public interests and concerns, enabling them to provide more targeted and reliable health care services.

## Introduction

Cancer poses a significant burden on global public health [1]. In urology, bladder, kidney, and prostate cancers are the 3 primary genitourinary cancers contributing to the global burden of disease (GBD), with persistently high morbidity and mortality rates [1,2]. According to a GBD report from 2019, the annual global incidence rates of bladder, kidney, and prostate cancers have risen by 154.78%, 123.34%, and 169.11%, respectively, over the past 2 decades, making them the most prominent cancers in the field of urology [3,4]. In mainland China, the incidence rates of bladder, kidney, and prostate cancers in 2019 had doubled compared with 1990 and are projected to triple by 2030 [2], with 192,390 cases of bladder cancer, 126,980 cases of kidney cancer, and 315,310 cases of prostate cancer. Therefore, precautionary measures are essential in addition to gathering information and assessing the disease burden using real-world data.

Infodemiology was defined as “The framework for an emerging set of public health informatics methods to analyze search, communication and publication behavior on the Internet.” It has been shown to effectively highlight public health issues, assess the impact of health care policies, and uncover public concerns during global pandemics, as well as in chronic and contagious diseases, along with related public acceptance [5-9]. Examining underlying trends in user behavior and the specific demands associated with major genitourinary cancers could potentially provide insights into regional health information–seeking behaviors and population-level interests.

Cancer imposes a significant burden on patients and their families, typically measured in terms of financial costs and clinical outcomes [10,11]. However, there is limited understanding of its broader impacts, such as public awareness, emotional well-being, and social participation. In China, previous studies have examined prevalent noncancer urological issues and their online visibility using data from the Baidu Index [6,12,13], a tool that analyzes search behaviors to reflect public interest in health topics. While prior research has explored general cancer-related searches, these studies primarily focused on incidence correlations and population-level disparities across 28 cancer types [14], offering limited insights into specific user demands or temporal and geographic patterns.

This study aims to address this gap by focusing on 3 major genitourinary cancers: bladder cancer, kidney cancer, and prostate cancer. Using Baidu Index data, we analyzed internet search trends, user needs, and associated geospatial and temporal patterns. By identifying search behaviors and topics of interest, we seek to provide actionable insights into public health awareness and address unmet needs, potentially contributing to the improvement and guidance of health care strategies.

## Methods

### Keyword Selecting and Data Retrieving

Baidu (Baidu, Inc.), the leading search engine in mainland China, accounts for 92.1% of the search volume and 93.1% of user coverage [15]. Its analytics platform, Baidu Index, allows for tracking keyword popularity trends and analyzing related user demands [6,7,14]. Comparable to Google’s platform on a global scale, Baidu has been validated as a reliable tool for studying online search trends and user behavior in infodemiology research within China [16,17].

This study primarily focuses on analyzing the temporal search trends of cancer-related terms associated with kidney, bladder, and prostate cancers. Based on defined criteria, these terms are characterized as compounds [6], combining morphemes denoting a urological organ with those indicating tumor-related concepts. The key morphemes identified were (1) “肾脏” or “肾” (the kidney), (2) “膀胱” (the bladder), (3) “前列腺” (the prostate), and (4) “肿瘤” (the tumor). The Baidu Index platform automatically matched these combinations with all available search keywords, including synonyms and complex derivatives. Measures were implemented to prevent duplication and omissions, following approaches detailed in previous studies [6,18]. Synonyms and complex derivatives were screened and selected to minimize ambiguity and bias arising from language habits, as previously described [6,13]. All available search keywords related to these 3 cancer types were categorized based on their connotations and are listed in Multimedia Appendix 1.

The Baidu Index platform consists of 3 key modules: (1) the search trend module, (2) the user demand module, and (3) the demographic portrait module. These modules enable the analysis of search demand from multiple perspectives, including popularity trends, topic-related concerns, and geodemographic features [6,13]. Search popularity is quantified using the Baidu Search Index (BSI), a key metric based on daily recorded search demand. With integrated data on location, gender, age, and other elements, trends and demographic profiles of the population can be visualized and retrieved [6,13]. Search trend data, available since 2011, were retrieved from the search trend module of the Baidu Index platform [14]. Data at both provincial and national scales were collected for the period from January 1, 2011, to July 31, 2023. The most recent data from the geodemographic and user demand modules were obtained from the user demand module on the Baidu Index official website [9,19].

### Data Processing and Statistical Analysis

The Baidu Index is a data-sharing platform that leverages extensive user behavior data to measure search trends. By tracking the frequency of unique keyword searches and their weighting within Baidu’s overall search volume, it provides a metric for keyword popularity. This study collected data on a daily, weekly, monthly, and yearly basis to capture a comprehensive view of cancer-related search patterns. Sequential plotting of BSI data for each term was conducted to illustrate trends in public interest. Changes in trends over time were analyzed using the Joinpoint Regression Model (program version 4.7.0.0; Statistical Research and Applications Branch, National Cancer Institute). This model, well-suited for time-series analysis of large data sets, identifies statistically significant shifts in trends. The annual percentage change (APC) was calculated to summarize yearly trends within specified intervals, measuring year-over-year percentage changes. The average APC (AAPC) was used to evaluate trends over extended periods, providing a more stable estimate of the overall trend direction and rate of change [20,21]. For each topic—bladder cancer, kidney cancer, and prostate cancer—the public demand trend was illustrated through sequentially plotted BSI data. Intergroup differences were analyzed using the Student *t* test and Kruskal-Wallis test, as appropriate. A *P* value of <.05 was considered statistically significant.

In the user demand section, the top 10 most frequently mentioned words related to each search keyword were listed weekly and sorted by cancer type. This allowed for the identification and analysis of the most prominent and commonly discussed topics for each cancer type. In line with previous findings, we used a 13-topic system to categorize user demand–related terms, helping to clarify users’ main concerns and implied intentions [13]. Aside from some random or off-topic terms, these categories were defined as follows: (1) complaint, (2) inquiry, (3) treatment and decision, (4) health issue, (5) diagnosis, (6) hospital and service, (7) symptom confirmation, (8) tests and examinations, (9) prognosis, (10) traditional Chinese medicine (TCM) complaint, (11) TCM inquiry, (12) TCM ingredient, and (13) TCM regimen.

All databases were constructed using Excel 2019 (Microsoft Corporation). The APC was calculated with the Joinpoint Regression Model, program version 4.7.0.0 (Statistical Research and Applications Branch, National Cancer Institute). Statistical analysis and figure creation were performed using Prism 9 for macOS (version 9.5.0 (525); GraphPad Software).

### Ethical Considerations

We used publicly available, anonymized data that can be accessed without special permissions. As the data are aggregated and publicly accessible, IRB approval or exemption was not required.

## Results

### Available Trends Data in Urology Cancer Topics

We identified and confirmed 39 valid search keywords on the Baidu Index platform. These keywords are theme-based synonyms and moderate derivative terms that convey specific motives or demands. Among these, 13 keywords pertained specifically to bladder cancer, while 15 and 11 keywords were related to kidney cancer and prostate cancer, respectively. For theme categorization, 4 topics were identified: (1) complaint, with 9 keywords; (2) inquiry, with 23 keywords; (3) treatment, with 4 keywords; and (4) prognosis, with 3 keywords. All available search keywords related to these 3 urological cancers, along with their English equivalent translations, are listed in Multimedia Appendix 1.

**Figure 1:**
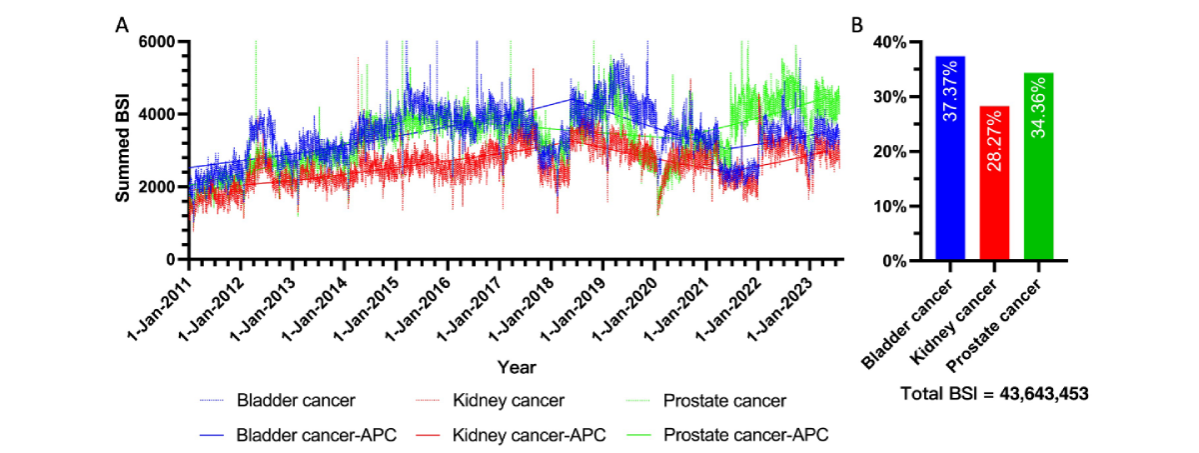
Online search trends in bladder, kidney, and prostate cancer topics since 2011. (A) Searching trend of each cancer topic; (B) Sum BSI proportion of each cancer topic. APC: annual percentage change; BSI: Baidu Search Index.

**Figure 2:**
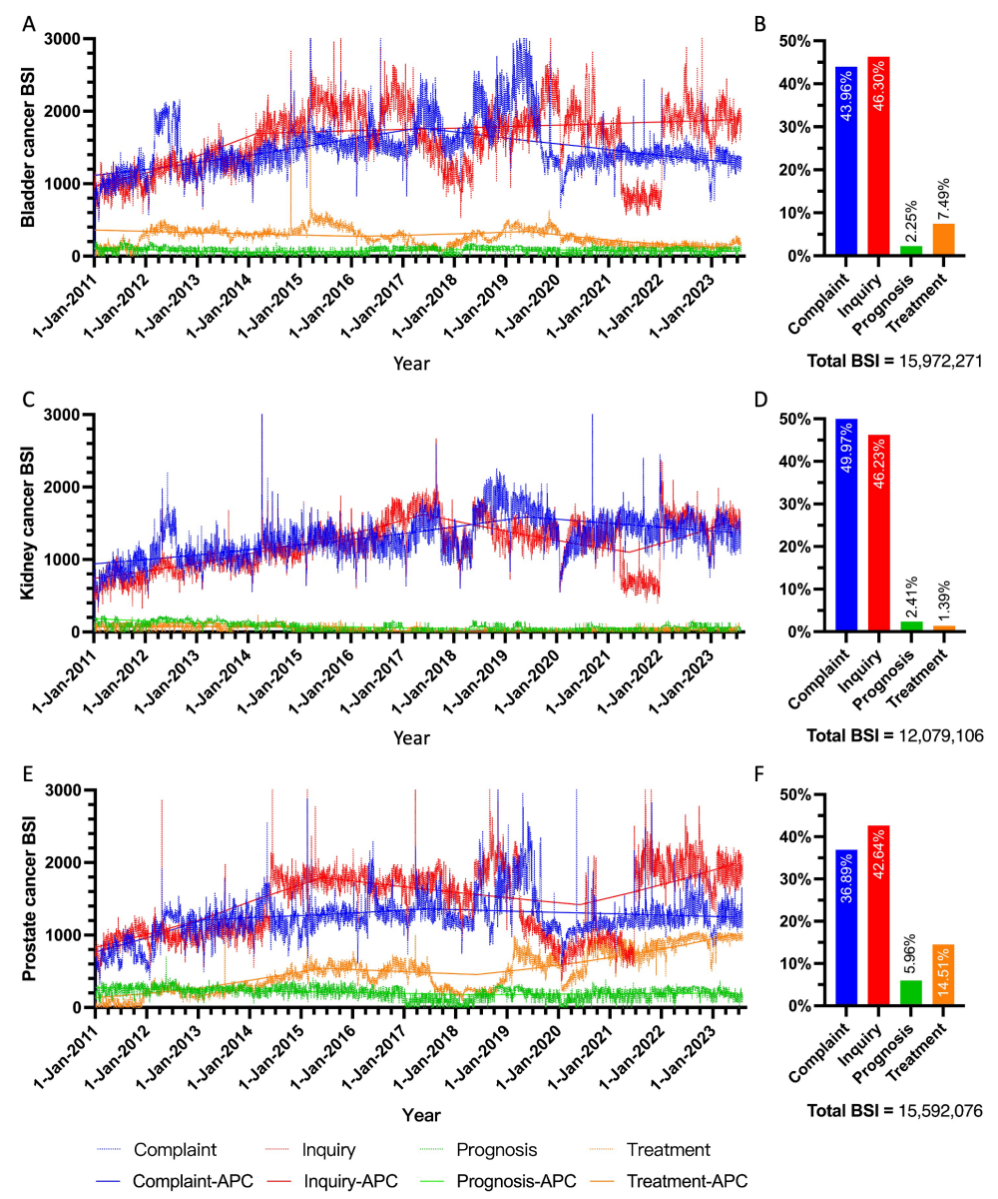
Online search trends for specific topics related to bladder, kidney, and prostate cancer since 2011. (A) Searching trend of specific topics in bladder cancer. (B) Sum BSI proportion of specific topics in bladder cancer. (C) Searching trend of specific topics in kidney cancer. (D) Sum BSI proportion of specific topics in kidney cancer. (E) Searching trend of specific topics in prostate cancer. (F) Sum BSI proportion of specific topics in prostate cancer. APC: annual percentage change; BSI: Baidu Search Index.

The general search volume for all 3 urological cancers increased to a mean of 10,737.74 (SD 1026.29) from an initial mean of 5975.68 (SD 770.42). Specifically, the average daily search volume for bladder cancer rose to 3453.09 (SD 337.44) in 2023 from an initial average of 2275.72 (SD 302.17). For kidney cancer, the average daily search volume increased to 2976.78 (SD 319.64) from an initial average of 1706.84 (SD 262.95). Similarly, the search volume for prostate cancer grew to a mean of 4307.87 (SD 417.68) from 1993.12 (SD 297.99). According to the trend module, the total BSI for these top 3 urological cancers was 43,643,453. Specifically, the 13-year summed BSI ratio was 37.37% (15,972,271/43,643,453) for bladder cancer, 28.27% (12,079,106/43,643,453) for kidney cancer, and 34.36% (15,592,076/43,643,453) for prostate cancer (Figure 1). Regarding topic preferences for each cancer, the BSI ratio for complaint and inquiry was dominant, accounting for 90.26%, 96.10%, and 79.53% across all 4 topics for bladder cancer, kidney cancer, and prostate cancer, respectively (Figure 2).

To illustrate search trends over time since January 1, 2011, the daily request–based BSI for each cancer was analyzed both overall and by specific topics. The significance of these trends was evaluated using the APC model, as shown in Figures 1 and 2.

Based on the average annual BSI counts, a general growth in search requests for all 3 urological cancers was observed. The overall APC was 15.2% (*P*<.05) from 2011 to 2015, –2.8% from 2015 to 2021, and 8.9% from 2021 to 2023, resulting in an AAPC of 4.9%. For bladder cancer, the APC was 8.3% (*P*<.05) from 2011 to 2019, –11.7% from 2019 to 2021, and 7.4% from 2021 to 2023, with an AAPC of 2.8%. For kidney cancer, the APC was 8.0% (*P*<.05) from 2011 to 2019, –9.6% from 2019 to 2021, and 11.4% from 2021 to 2023, resulting in an AAPC of 3.9%. For prostate cancer, the APC was 17.7% (*P*<.05) from 2011 to 2015, –3.1% from 2015 to 2020, and 10.4% from 2021 to 2023, yielding an AAPC of 6.8% (*P*<.05).

Specifically within the bladder cancer theme, the APCs for the *complaint* topic were 8.0% (*P*<.05) from 2011 to 2021 and –5.1% from 2021 to 2023, with an AAPC of 1.2%. For the *inquiry* topic, the APCs were 20.8% (*P*<.05) from 2011 to 2014 and 1.2% from 2014 to 2023, with an AAPC of 4.8% (*P*<.05). For the *prognosis* topic, the APCs were –20.8% (*P*<.05) from 2011 to 2014, 15.6% from 2014 to 2018, and –6.9% from 2018 to 2023, resulting in an AAPC of –4.4%. For the *treatment* topic, the APCs were –5.4% from 2011 to 2016, 7.6% from 2016 to 2019, and –23.9% (*P*<.05) from 2019 to 2023, with an AAPC of –9.4%.

In the kidney cancer theme, the APCs for the *complaint* topic were 6.8% (*P*<.05) from 2011 to 2019 and –3.6% from 2019 to 2023, with an AAPC of 3.2% (*P*<.05). For the *inquiry* topic, the APCs were 14.0% (*P*<.05) from 2011 to 2017, –9.2% (*P*<.05) from 2017 to 2021, and 17.8% from 2021 to 2023, resulting in an AAPC of 6.2% (*P*<.05). For the *prognosis* topic, the APCs were –19.0% (*P*<.05) from 2011 to 2019 and 15.7% from 2019 to 2023, with an AAPC of –11.0% (*P*<.05). For the *treatment* topic, the APCs were 2.5% from 2011 to 2014, –47.1% from 2014 to 2018, and 42.6% from 2019 to 2023, with an AAPC of –12.2% (*P*<.05). In the prostate cancer theme, the APCs for the *complaint* topic were 25.4% (*P*<.05) from 2011 to 2013, 3.1% from 2013 to 2017, and –1.4% from 2017 to 2023, resulting in an AAPC of 4.2%. For the *inquiry* topic, the APCs were 21.2% (*P*<.05) from 2011 to 2015, –4.6% from 2015 to 2020, and 11.8% from 2020 to 2023, with an AAPC of 11.8%. For the *prognosis* topic, the APCs were –6.2% (*P*<.05) from 2011 to 2018 and 2.5% from 2018 to 2023, with an AAPC of –3.0% (*P*<.05). For the *treatment* topic, the APCs were 41.6% (*P*<.05) from 2011 to 2015, –5.8% from 2015 to 2018, and 17.4% (*P*<.05) from 2018 to 2023, resulting in an AAPC of 18.2% (*P*<.05).

### Geographic Differences

**Figure 3:**
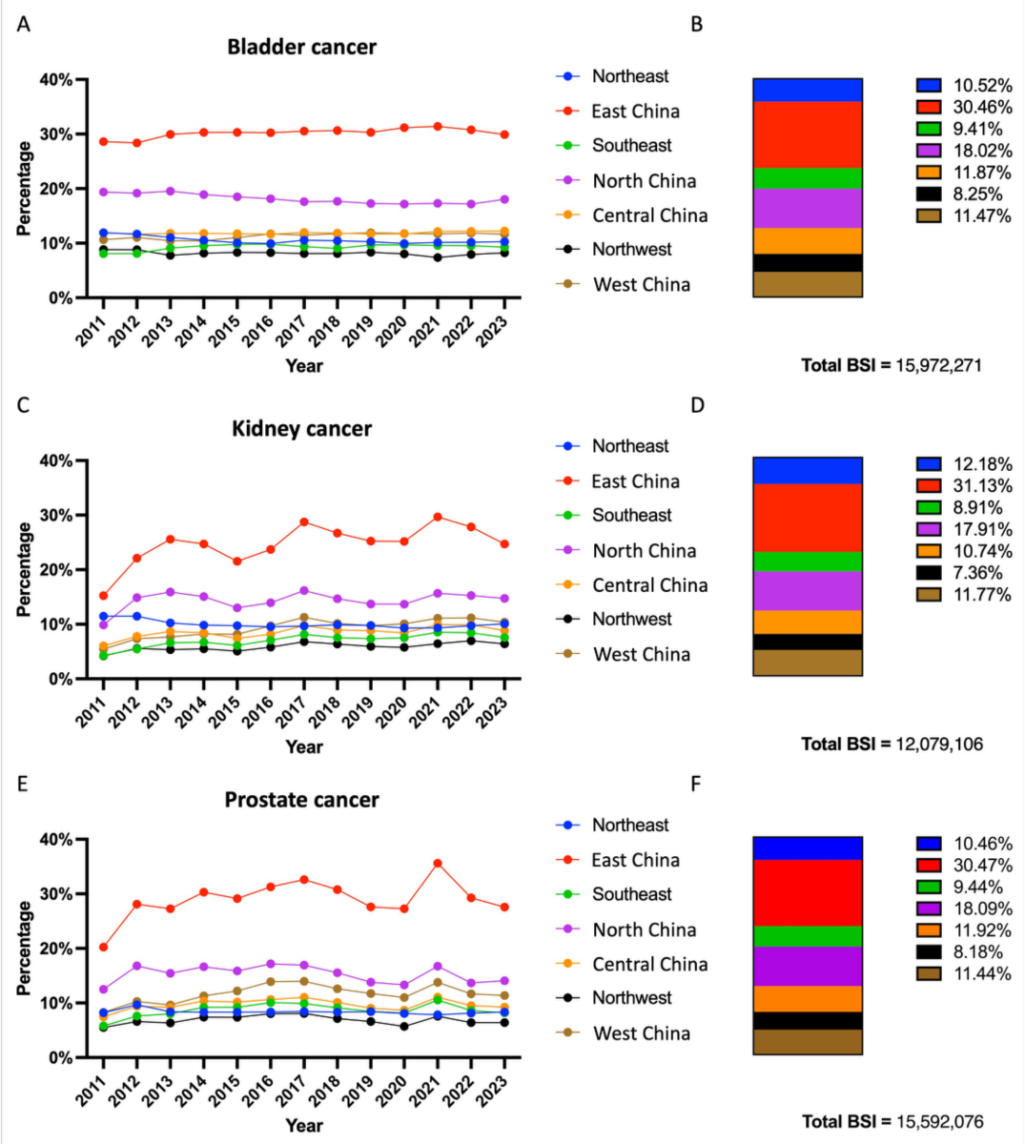
Regional distribution of online search in bladder, kidney, and prostate cancer topics. (A) Annual BSI trend for each region in the topic of bladder cancer. (B) Total search rates for each region on the topic of bladder cancer. (C) Annual BSI trend for each region in the topic of kidney cancer. (D) Total search rates for each region on the topic of kidney cancer. (E) Annual BSI trend for each region in the topic of prostate cancer. (F) Total search rates for each region on the topic of prostate cancer. BSI: Baidu Search Index.

The geographic distribution of each cancer type was calculated based on provincial BSI data and categorized according to the 7 Chinese administrative divisions [6]. Figure 3 shows the 13-year regional BSI proportions for each cancer type with valid search records. Search requests were predominantly from East China, accounting for 30.46%, 31.13%, and 30.47% of bladder, kidney, and prostate cancer searches, respectively, followed by North China. Search demand from Central China, South China, and West China was comparable, each contributing around 11%. The Northeast and Northwest regions ranked the lowest, collectively accounting for approximately 8% of searches for each urological cancer.

### Demographic Differences

From the demographic distribution analysis, variations in age and gender distribution were observed across each cancer theme and specific topic. In the bladder cancer theme, search requests for each topic were primarily made by individuals aged 20-29 and 30-39 years. For kidney cancer, the topics of complaint, inquiry, and prognosis were predominantly searched by the 20-29-year age group, whereas the topic of treatment was mainly searched by individuals aged 40-49 years. In the prostate cancer theme, the topics of inquiry and treatment were primarily requested by the 30-39-year age group, while searches for prognosis were mainly made by those aged 40-49 years. Notably, no dominant age group was identified for searches in the complaint topic for prostate cancer (Table 1).

**Table 1:** Demographic differences in each cancer topic.

Theme and topic		≤19 years	20-29 years	30-39 years	40-49 years	≥50 years	Female	Male
**Bladder cancer**								
	Complaint, n/N (%)	2447/40,701 (6.01)	12,411/40,701 (30.49)	14,187/40,701 (34.86)	6954/40,701 (17.09)	4702/40,701 (11.55)	22,332/40,701 (54.87)	18,369/40,701 (45.13)
	Enquiry, n/N (%)	2149/58,805 (3.65)	10,237/58,805 (17.41)	18,226/58,805 (30.99)	15,246/58,805 (25.93)	12,947/58,805 (22.02)	34,729/58,805 (59.06)	24,076/58,805 (40.94)
	Treatment, n/N (%)	45/2567 (1.75)	907/2567 (35.33)	730/2567 (28.44)	486/2567 (18.93)	398/2567 (15.50)	1195/2567 (46.55)	1371/2567 (53.41)
	Prognosis, n/N (%)	256/6017 (4.25)	1298/6017 (21.57)	2217/6017 (36.85)	1245/6017 (20.69)	1001/6017 (16.64)	3342/6017 (55.54)	2675/6017 (44.46)
**Kidney cancer**								
	Complaint, n/N (%)	2548/40,181 (6.34)	12,059/40,181 (30.01)	14,170/40,181 (35.27)	7312/40,181 (18.20)	4092/40,181 (10.18)	20,819/40,181 (51.81)	19,362/40,181 (48.19)
	Enquiry, n/N (%)	1927/49,219 (3.92)	9211/49,219 (18.71)	15,897/49,219 (32.30)	12,657/49,219 (25.72)	9527/49,219 (19.36)	28,829/49,219 (58.57)	20,390/49,219 (41.43)
	Treatment, n/N (%)	0/1328 (0.00)	221/1328 (16.64)	296/1328 (22.29)	738/1328 (55.57)	73/1328 (5.50)	664/1328 (50.00)	664/1328 (50.00)
	Prognosis, n/N (%)	0/1913 (0.00)	201/1913 (10.51)	705/1913 (36.85)	453/1913 (23.68)	554/1913 (28.96)	1325/1913 (69.26)	588/1913 (30.74)
**Prostate cancer**								
	Complaint, n/N (%)	2011/39,725 (5.06)	8413/39,725 (21.18)	10,388/39,725 (26.15)	8457/39,725 (21.29)	10,456/39,725 (26.32)	17,602/39,725 (44.31)	22,123/39,725 (55.69)
	Enquiry, n/N (%)	3786/59,541 (6.36)	14,445/59,541 (24.26)	20,435/59,541 (34.32)	12,156/59,541 (20.42)	8719/59,541 (14.64)	27,446/59,541 (46.10)	32,095/59,541 (53.90)
	Treatment, n/N (%)	116/4462 (2.60)	1007/4462 (22.57)	1344/4462 (30.12)	1063/4462 (23.82)	932/4462 (20.89)	2053/4462 (46.01)	2409/4462 (53.99)
	Prognosis, n/N (%)	833/30,625 (2.72)	3479/30,625 (11.36)	8780/30,625 (28.67)	9460/30,625 (30.89)	8073/30,625 (26.36)	16,363/30,625 (53.43)	14,262/30,625 (46.57)

### Keywords, Related Terms, and Search Frequency

**Figure 4:**
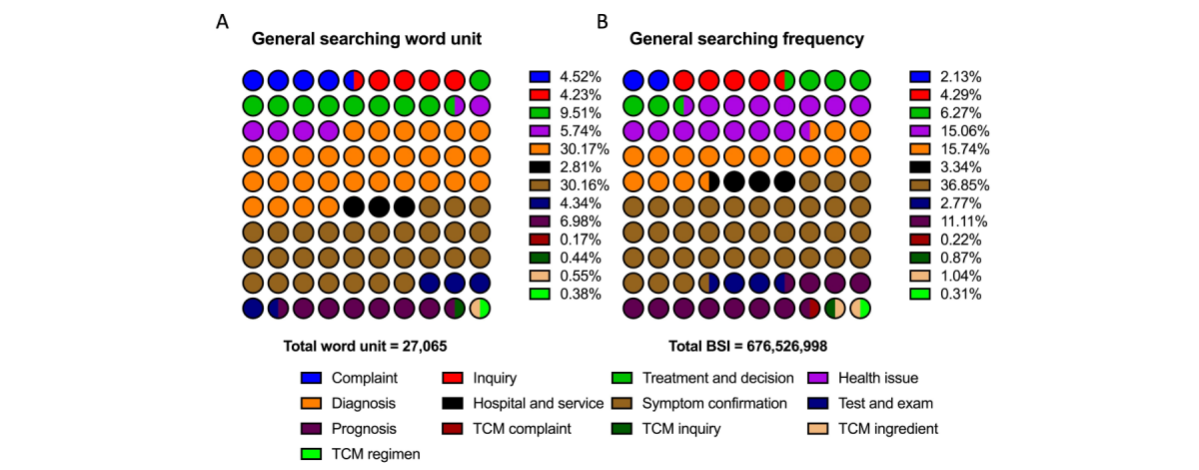
erm categories related to all cancers (bladder, kidney, and prostate) in the Baidu Index user demand module (August 2022 to August 2023). (A) The most frequently appearing related words (word units) in Baidu Index searches related to bladder, kidney, and prostate cancers. (B) The most searched related words in Baidu Index inquiries related to bladder, kidney, and prostate cancers. BSI: Baidu Search Index; TCM: traditional Chinese medicine.

**Figure 5:**
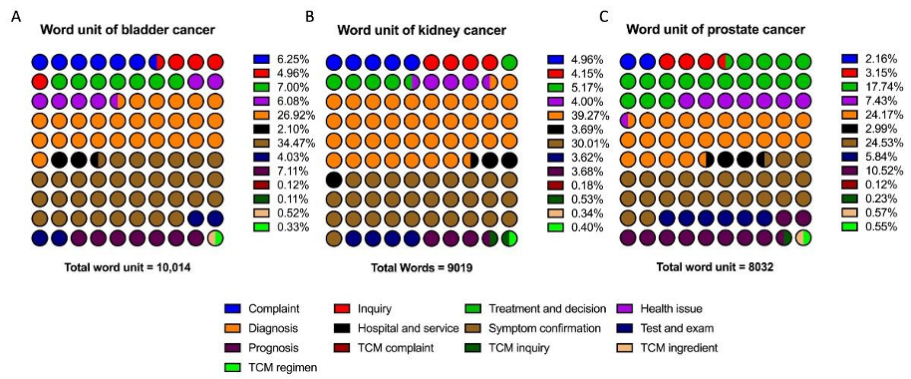
Term categories of the most frequently appearing related words for each cancer (August 2022 to August 2023). (A) Most frequently appearing related words (word units) in Baidu Index searches related to (A) bladder cancer, (B) kidney cancer, and (C) prostate cancer. TCM: traditional Chinese medicine.

**Figure 6:**
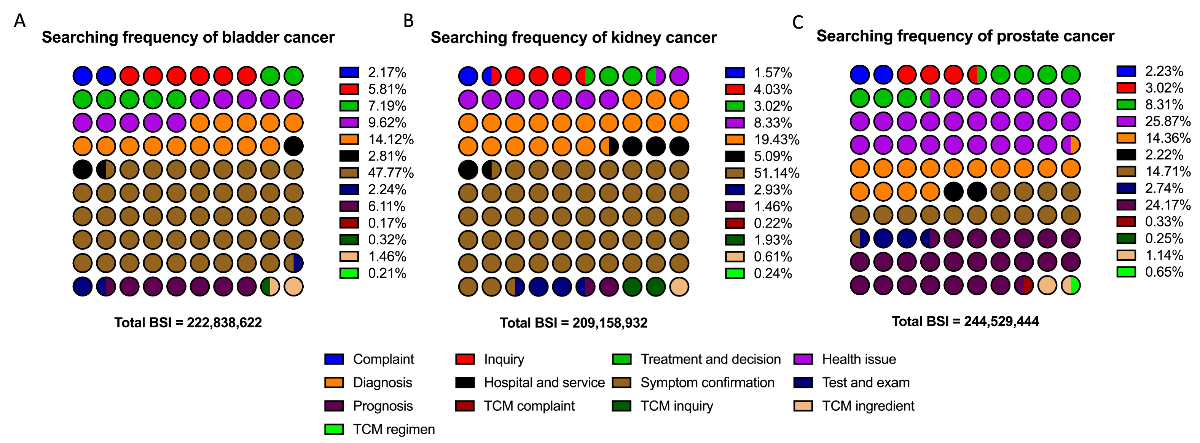
Term categories related to the most searched related words for each cancer (August 2022 to August 2023). Most searched related words (word units) in Baidu Index searches related to (A) bladder cancer, (B) kidney cancer, and (C) prostate cancer. BSI: Baidu Search Index; TCM: traditional Chinese medicine.

During the data-providing period from August 15, 2022, to August 13, 2023, 27,065 out of 31,200 words were identified as in-topic, representing valid user demand. The total BSI for these relevant user demand terms was 676,526,998, accounting for only 676,526,998 of 2,570,697,380 (15.74%) total search requests. Detailed distributions of these relevant terms and their search frequencies are shown in Figure 4. The valid search ratio and demand distribution were also summarized overall (Figure 4) and specifically for each cancer theme (Figures 5 and 6). Additionally, the 3 most representative user-demand issues were identified and ranked based on frequency and search popularity (Tables 2 and 3).

**Table 2:** The top 3 most frequently appearing related words (word units) searched in the Baidu Index for each type of cancer.

Category and term			Bladder cancer		Count, n		Kidney cancer		Count, n		Prostate cancer		Count, n
**Complain**													
	Term 1	血尿(Hematuria)		96		血尿 (Hematuria)		51		尿潴留 (Urinary retention)		19	
	Term 2	尿血 (Urinate with blood)		79		无痛血尿 (Painless hematuria)		40		血精 (Hemospermia)		9	
	Term 3	无痛血尿 (Painless hematuria)		74		尿血 (Urinate with blood)		33		前列腺痛 (Prostatic pain)		7	
**Etiology and causes**													
	Term 1	小便时尿出血是怎么回事 (What is urinate with blood)		81		肾肿瘤分类 (Phenotypes in kidney cancer)		19		前列腺钙化是什么意思 (What is prostatic calcification)		34	
	Term 2	尿频尿急尿不尽是什么原因造成的 (What caused urinary frequency, urgency, and incomplete urinate)		26		肾积水是什么原因造成的怎么治疗 (What caused the hydronephrosis)		13		前列腺钙化灶是什么意思 (What does prostatic calcification mean)		26	
	Term 3	尿频繁是什么原因 (What caused frequently urination)		19		小便时尿出淡红色是怎么回事 (What caused reddish urination)		10		前列腺炎怎么引起的 (What caused prostatitis)		11	
**Treatment and pharmaceutical**													
	Term 1	膀胱癌治疗 (Treatment for bladder cancer)		115		肾癌的治疗 (Treatment for kidney cancer)		19		前列腺癌治疗 (Treatment for prostate cancer)		129	
	Term 2	膀胱炎怎么治疗 (Treatment for cystitis)		21		肾囊肿怎么治疗 (Treatment for renal cyst)		11		前列腺增生的最佳治疗方法 (Best treatment for prostate cancer)		96	
	Term 3	前列腺癌治疗 (Treatment for prostate cancer)		13		肾肿瘤切除 (Nephrectomy for kidney cancer)		10		前列腺癌的治疗 (Prostate cancer treatment)		74	
**Health care–related terms**													
	Term 1	膀胱 (Bladder)		169		肿瘤 (Tumor)		24		前列腺 (Prostate)		174	
	Term 2	四种癌已经不是癌了 (4 no longer diseases defined as cancer)		117		肾脏 (Kidney)		23		四种癌已经不是癌了 (4 no longer diseases defined as Cancer)		165	
	Term 3	芳香胺 (Aromatic amines)		28		肾癌饮食 (Diet for kidney)		17		前例腺 (“Prastate”)		7	
**Diagnosis**													
	Term 1	膀胱癌 (Bladder cancer)		499		肾癌 (Kidney cancer)		395		前列腺癌 (Prostate cancer)		422	
	Term 2	膀胱肿瘤 (Bladder tumor)		180		肾囊肿 (Renal cyst)		242		前列腺癌骨转移 (Prostate cancer with bone metastasis)		115	
	Term 3	膀胱炎 (Cystitis)		169		肾肿瘤 (Tumors in kidney)		230		前列腺炎 (Prostatitis)		108	
**Health care services and commodities**													
	Term 1	北京大学第一医院 (The first affiliate Hospital of PKU)		4		中国最好的肾病医院 (The best hospital for treating kidney diseases)		42		海外医疗 (Oversee medication service)		15	
	Term 2	泌尿外科哪个医院好 (What is the best urology hospital)		4		治疗肾癌最好的医院 (The best hospital for treating kidney cancers)		30		麻省总医院 (Massachusetts General Hospital)		14	
	Term 3	吴阶平 (Prof Jiepin Wu)		3		肿瘤医院全国排名一 (National NO.1 Oncology Hospital)		10		厚朴方舟 (Hopenoak.com)		7	
**Diagnosis confirmation**													
	Term 1	膀胱癌早期是什么症状 (Early symptoms of bladder cancer)		431		肾癌早期的五个表现 (Early symptoms of bladder cancer)		376		前列腺癌症状有哪些 (What are the symptoms of prostate cancer)		356	
	Term 2	膀胱炎是什么症状表现 (What are the symptoms of cystitis)		204		肾癌症状 (Symptoms of kidney cancer)		134		前列腺癌症状 (Symptoms of prostate cancer)		149	
	Term 3	膀胱癌症状 (Symptoms of bladder cancer)		202		肾衰竭的早期症状表现 (Early symptoms of kidney cancer)		123		前列腺癌的症状 (Prostate cancer symptoms)		226	
**Test and examination**													
	Term 1	膀胱镜 (Cystoscope)		140		肾钙化 (Renal calcification)		12		前列腺特异性抗原 (Prostate specific antigen)		98	
	Term 2	膀胱检查 (Bladder examination)		19		肾功能检查哪些项目 (Items of renal function test)		10		PSA (PSA)		72	
	Term 3	尿常规能检查出什么 (What can routine urinary test tell)		10		肾穿刺 (Renal puncturing)		10		前列腺炎一杯水自测 (Confirming prostatitis with a cup of water)		30	
**Prognosis**													
	Term 1	膀胱癌能活多久 (How long one can live with diagnosed bladder cancer)		210		肾癌晚期能活多久 (How long one can live with diagnosed late-stage kidney cancer)		51		前列腺癌能活多久 (How long one can live with diagnosed prostate cancer)		342	
	Term 2	膀胱癌晚期 (Late-stage bladder cancer)		125		晚期肾癌 (Late-stage kidney cancer)		17		前列腺炎有什么症状和危害性 (Symptoms and hazard of prostatitis)		213	
	Term 3	前列腺癌能活多久 (How long one can live with diagnosed prostate cancer)		67		肺癌晚期能活多久 (How long one can live with diagnosed late-stage lung cancer)		16		前列腺癌晚期 (Late-stage prostate cancer)		122	
**TCM^a^ diagnosis**													
	Term 1	疾在腠理 (Disease sign on the skin)		1		肾虚 (Insufficiency in “Shen” essence)		3		湿热症疹状 (Rashes of the humid heat symptoms)		2	
	Term 2	肾精亏耗 (Depletion of “Shen” essence)		1		肾阳虚 (Insufficiency in “Shen” essence of “Yang”)		2		五心烦热 (Sphoria with feverish sensation in chest, palms, and soles)		1	
	Term 3	肾虚 (Insufficiency in “Shen” essence)		1		肾阴虚 (Insufficiency in “Shen” essence of “Yin”)		2		湿热疹 (Rashes of the humid heat)		1	
**TCM diagnosis confirmation**													
	Term 1	肾虚的表现症状有哪些 (What are the symptoms of insufficiency in “Shen” essence)		3		肾虚的表现症状有哪些 (What are the symptoms of insufficiency in “Shen” essence)		21		中医治疗前列腺 (TCM treatment of prostate gland)		2	
	Term 2	肾虚的症状 (Symptoms of insufficiency in “Shen” essence)		2		如何保养肾 (How to maintain kidney with “Shen” essence)		2		中医治疗癌症 (TCM treatment of cancer)		1	
	Term 3	拔罐的好处与功效 (Benefit and efficacy of cupping cup)		1		肾俞穴 (Acupoint of “Shen Yu”)		2		中医治疗肿瘤 (TCM treatment of tumor)		1	
**TCM regimen**													
	Term 1	五味子 (*Schisandra chinensis* Turcz. Baill.)		2		丝瓜子 (Loofah seed)		1		车前草 (Plantago asiatica L.)		2	
	Term 2	黑枸杞的作用与功效 (The efficacy of Lycium ruthenicum Murr)		2		丝瓜的功效与作用禁忌 (The efficacy and contraindication of loofah)		1		三七的副作用太大了 (The side effect of Panax pseudo-ginsenga)		1	
	Term 3	东革阿里的功效 (The efficacy of Tongkat Ali)		2		丝瓜蒂 (*Luffa cylindrica* （L.）Roem.)		1		东阿阿胶250克价格 (Price of donkey-hide gelatin)		1	
**TCM remedy and materials**													
	Term 1	银花泌炎灵片 (Tablet of “YinHuaMiYanLin”)		3		云南白药气雾剂的作用与功效 (The efficacy of “Yunnan Baiyao” spray)		1		金水宝胶囊的功效与主治 (The efficacy and indications of “JinshuiBao” caspule)		3	
	Term 2	仙鹤神针 (The miraculous needle of “Crane”)		2		加味二陈汤 (Potion of two old ingredient with extra additional)		1		抗肿瘤最强的中草药 (The strongest anti-tumor TCM herb)		2	
	Term 3	桂枝茯苓丸 (Guizhi Ling Pills)		2		华蟾素 (Cinobufagin)		1		乌头赤石脂丸 (Aconite red halloysite)		1	

^a^TCM: traditional Chinese medicine.

**Table 3:** The top 3 most searched related words in the Baidu Index for each type of cancer.

Category and terms			Bladder cancer		Baidu Search Index		Renal cancer		Baidu Search Index		Prostate cancer		Baidu Search Index
**Complain**													
	Term 1	尿血 (Urinate with blood)		11,24,546		尿隐血 (Occult hematuria)		5,68,694		白肺 (“White Lung”)		36,90,652	
	Term 2	血尿 (Hematuria)		9,34,444		血尿 (Hematuria)		4,91,164		尿潴留 (Urinary retention)		2,08,682	
	Term 3	小便尿完过一会又想尿 (Small moment of urinate urge after pee)		5,17,276		尿血 (Urinate blood)		4,82,462		阳痿 (Impotence)		1,46,838	
**Etiology and causes**													
	Term 1	尿频尿急尿不尽是什么原因造成的 (What caused urinary frequency, urgency, and incomplete urinate)		25,14,540		白肺是什么意思 (What does the “White Lung” mean)		7,60,196		前列腺钙化是什么意思 (What does prostate calcification mean)		11,11,802	
	Term 2	小便时尿出血是怎么回事 (Why is urinate with blood)		24,30,620		尿酸高是什么引起的原因 (What causes hyperuricemia)		5,51,308		前列腺钙化灶是什么意思 (What is prostatic calcification)		10,02,476	
	Term 3	尿频繁是什么原因 (What causes frequently urination)		11,30,904		尿液发红褐色怎么回事 (What causes reddish urine)		4,84,088		前列腺炎怎么引起的 (What causes prostatitis)		8,72,092	
**Treatment and pharmaceutical**													
	Term 1	甲流吃什么药效果最好 (What is the medication of Influenza A virus)		19,31,970		肾结石怎么排出来最快方法 (The fastest way of urinate out the kidney stone)		4,53,232		前列腺增生的最佳治疗方法 (The best way of treating BPH)		51,31,986	
	Term 2	布洛芬混悬液 (Ibuprofen Suspension)		18,84,472		靶向治疗是什么意思 (What does the targeted therapy mean)		2,30,322		前列腺增生的症状 (Symptoms of BPH)		18,04,768	
	Term 3	尿路感染10分钟解决方法 (Method of eradicate UTI within 10 minutes)		10,87,080		腰椎间盘突出最好的治疗方法 (Best treatment for lumber disc protrusion )		1,88,922		前列腺炎吃什么药效果好见效快 (What medication for prostatitis effect promptly)		11,47,908	
**Health care–related terms**													
	Term 1	中国知网 (CKNI.COM)		35,69,116		中国知网 (CKNI.COM)		72,65,752		前列腺 (Prostatitis)		1,46,50,248	
	Term 2	四种癌已经不是癌了 (4 no longer cancer defined cancers)		30,23,448		咸阳疫情最新消息 (The latest pandemic news in Xianyang City)		6,48,786		四种癌已经不是癌了 (4 no longer cancer defined cancers)		43,33,372	
	Term 3	膀胱 Bladder()		25,49,522		肿瘤 (Tumor)		4,80,378		中国知网 (CKNI.COM)		22,87,492	
**Diagnosis**													
	Term 1	膀胱癌 (Bladder caner)		59,88,140		肾囊肿 (Renal cyst )		91,41,308		前列腺炎 (Prostatitis)		1,20,84,428	
	Term 2	膀胱炎 (Cystitis)		36,16,898		肾结石 (Nephrolithiasis)		36,13,030		前列腺癌 (Prostate cancer)		93,56,312	
	Term 3	前列腺癌 (Prostate cancer)		27,19,162		肾癌 (Kidney cancer)		32,98,004		前列腺增生 (Benign prostate hyperplasia)		22,11,278	
**Health care services and commodities**													
	Term 1	山西医科大学 (Shanxi Medical School)		4,62,288		北京大学 (Paikin University)		4,80,608		华中科技大学 (Huazhong University of Science and Technology)		7,48,616	
	Term 2	哈尔滨医科大学 (Harbin medical university)		4,17,328		吉林大学 (Jilin university)		2,53,870		男科医院 (Andrology hospital)		2,96,596	
	Term 3	问医生 (Ask Doctor.com)		1,86,720		复旦大学 (Fudan University)		2,07,778		百度健康 (Baidu Health)		2,61,804	
**Diagnosis confirmation**													
	Term 1	甲流感症状有哪些 (What are the symptoms of influenza A)		1,94,65,776		肾衰竭的早期症状表现 (Early symptoms of renal failure)		1,29,43,182		前列腺癌症状有哪些 (What are the symptoms of prostate cancer)		70,24,984	
	Term 2	膀胱炎是什么症状表现 (What are the symptoms of cystitis )		1,27,22,312		肾炎的症状是什么 (What are the symptoms of nephritis)		1,02,09,324		胰腺癌的早期症状 (What are the symptoms of pancreas cancer)		21,36,332	
	Term 3	膀胱癌早期是什么症状 (What are the early symptoms of bladder cancer)		86,79,854		尿毒症的早期症状 (What are the early symptoms of uremia)		95,76,026		如何判断自己前列腺炎 (How to determine prostatitis by my self)		21,03,682	
**Test and examination**													
	Term 1	血氧饱和度 (Blood oxygen saturation)		3,72,030		肌酐高是什么问题 (What are the problems causing high creatine level)		6,23,962		PSA (“PSA”)		7,98,870	
	Term 2	膀胱镜 (Cystoscope)		3,29,426		肾功能检查哪些项目 (What are the items of renal function test)		3,92,388		穿刺检查是什么意思 (What does “puncture & biopsy” means)		6,59,994	
	Term 3	血糖正常值 (Normal level of plasma glycose level)		2,99,716		血糖正常值 (Normal level of plasma glycose level)		2,92,004		前列腺炎一杯水自测 (Confirming prostatitis with a cup of water)		5,80,210	
**Prognosis**													
	Term 1	前列腺炎有什么症状和危害性 (Symptoms and hazard of prostatitis )		97,45,516		肺癌晚期能活多久 (How long one can live with late phase lung cancer)		8,41,568		前列腺炎有什么症状和危害性 (Symptoms and hazard of prostatitis)		5,15,38,700	
	Term 2	前列腺癌能活多久 (How long one can live with prostate cancer)		8,44,452		肺癌晚期最怕三个征兆 (The three poorest indications in late phase lung cancer)		3,65,816		前列腺癌能活多久 (How long one can live with prostate cancer)		42,52,848	
	Term 3	肺癌晚期能活多久 (How long one can live with late phase lung cancer)		7,57,840		不化疗和化疗哪个寿命长 (Chemo, or non-Chemo, which to choose for longer life)		2,77,808		白肺是可以治愈的吗 (Is “white lung” curable ())		10,53,438	
**TCM^a^ >diagnosis**													
	Term 1	舌苔发黄厚腻是什么原因怎么调理 (What causes thick and yellow tongue coating, how to moderate)		60,686		舌苔厚白是什么原因引起的怎么解决 (What causes thick and white tongue coating, how to moderate)		1,67,564		风热感冒和风寒感冒的症状区别 (Difference between “Feng heat” and “Feng cold” fever)		3,24,112	
	Term 2	飞机打多了属于阴虚还是阳虚 (Is too much masturbation causing essence deficiency in “Yin” or “Yang”)		33,180		肾阴虚 (Deficiency in “Shen” essence of “Yin”)		68,140		湿热疹 (Rash of humid heat)		2,01,220	
	Term 3	脾虚 (Deficiency in spleen essence)		24,362		肾阳虚 (Deficiency in “Shen” essence of “Yang”)		60,174		肾阴虚 (Deficiency in Shen” essence of “yin”)		33,262	
**TCM diagnosis confirmation**													
	Term 1	肾虚的表现症状有哪些 (What are the symptoms of deficiency in “Shen” essence)		6,83,020		肾虚的表现症状有哪些 (What are the symptoms of deficiency in “Shen” essence)		32,80,446		肾虚的表现症状有哪些 (What are the symptoms of deficiency in “Shen” essence)		1,66,796	
	Term 2	脾虚的表现和症状 (What are the symptoms of deficiency in “Pi” essence)		1,36,110		脾虚的表现和症状 (What are the symptoms of deficiency in “Pi” essence)		1,29,000		湿气重怎么排湿最有效 (How to expel humid “Qi” effectively when bearing too much humid “Qi”)		1,12,650	
	Term 3	肠胃不好怎么调理最有效 (How to moderate gastrointestinal function)		48,930		飞机打多了该怎么补回来 (How to compensate by supplement after loads of masturbation)		1,16,264		脾胃虚弱怎么调理最快 (What is the fastest way to moderate feeble “Pi and Wei”)		64,500	
**TCM regimen**													
	Term 1	金银花的功效与作用 (The efficacy of Honeysuckle)		5,70,726		山药的功效与作用 (The efficacy of Chinese yam)		2,01,052		茯苓的功效与作用 (The efficacy of poria)		3,72,518	
	Term 2	黑枸杞的作用与功效 (The efficacy of Lycium ruthenicum Murr)		3,86,932		甘草 (liquorice)		1,56,030		姜的功效与作用 (The efficacy of ginger)		2,59,726	
	Term 3	五味子 (Schisandra chinensis (Turcz.) Baill.)		2,83,100		蒲公英的功效与作用 (The efficacy of dandelion)		1,36,652		西洋参 (American ginseng)		2,08,448	
**TCM remedy and materials**													
	Term 1	右归丸的作用和功效 (The efficacy of “YouGuiWan” pill)		70,252		云南白药气雾剂的作用与功效 (The efficacy of “baiyao”， Yunnan)		41,140		六味地黄丸有什么功效与作用 (The efficacy of “LiuWeiDiHuangWan” pill)		3,43,428	
	Term 2	桂枝茯苓丸 (“GuizhiFulingWan” Pill)		62,076		四君子汤的功效与作用 (The efficacy of “Sijunzitang” potion)		32,698		金水宝胶囊的功效与主治 (The efficacy of “Jinshuibao” Capsule)		1,50,424	
	Term 3	龙胆泻肝丸 (“LongDanXieGanWan” Pill)		61,236		百令胶囊功效与作用 (The efficacy of “Bailing” Capsule)		30,136		玉屏风颗粒的功效与作用 (The efficacy of “Yupingfeng” electuary)		1,40,016	

^a^TCM: traditional Chinese medicine.

## Discussion

### Principal Findings

To the best of our knowledge, this study is the first infodemiology research to explore patients’ awareness and demand for primary urologic cancers (bladder, kidney, and prostate) within China’s vast population, particularly from clinical and health care perspectives [12,22]. By analyzing the most widely used local search platform, with billions of daily active queries, we identified consistent shifts in search trends, dominant regions for searches, and key demographic groups. Furthermore, we examined the most sought-after topics, reflecting user-initiated care-seeking behaviors and decision-making patterns [13].

We observed that while the overtime search trends fluctuate, the overall search volume for each cancer type has shown a general increase, as indicated by the overtime AAPC. Across all urologic cancer themes, the 4 main topics—“complaint,” “inquiry,” “treatment,” and “prognosis”—remain consistent, indicating that user queries focus on key aspects of disease-related decision-making. Notably, there is a strong demand for information on diagnostic criteria, etiology, treatment options, and realistic expectations for each cancer. Among tumor-related keywords, symptom-related searches account for 43.95%, 49.97%, and 36.89% of the total search volume for bladder, kidney, and prostate cancers, respectively. Inquiries, comprising 23 search keywords, account for 46.30%, 46.23%, and 42.64% of the total search volume for bladder, kidney, and prostate cancers, respectively. In terms of treatment-related searches, prostate cancer leads with 14.51% of the total search volume, followed by bladder cancer at 7.49% and kidney cancer at 2.41%. The prominence of searches related to complaints and inquiries is unsurprising, as the complaint category primarily includes diagnostic keywords, reflecting users’ initial, exploratory searches when they are uncertain about the specific information they require [22].

The main concerns in user inquiries revolve around early signs, primary indications, etiologies, specific symptoms, and other cancer-related issues. These topics represent a comprehensive collection of the most common challenges patients face during health care–seeking sessions, particularly in cancer-related scenarios.

In bladder cancer, search keywords frequently reflect concerns about symptoms, particularly in the early or late stages, with hematuria being a primary focus [23]. While hematuria does not definitively indicate bladder cancer, prompt attention to this visible symptom facilitates early detection and diagnosis.

For prostate cancer, user queries often center on symptoms, staging, and metastasis. Lower urinary tract symptoms are commonly reported but frequently stem from benign causes, making symptom-based identification challenging. Prostate cancer detection primarily relies on prostate-specific antigen-magnetic resonance imaging-biopsy combinations, though the screening sensitivity (0.93) and specificity (0.20) underscore limitations and adherence challenges [24-27]. Furthermore, concerns about skeletal metastasis and staging highlight the importance of enhanced education and communication strategies.

Kidney cancers, characterized by diverse carcinomas, are often identified through imaging as “space-occupying lesions,” resulting in ambiguous search terms like “mass (肿物).” Compared with bladder and prostate cancers, users frequently lack precise diagnostic information [28]. Advances in imaging technology have increased renal cell carcinoma detection rates by 3.1% annually over the past decade [29,30]. However, diagnostic accuracy remains limited by tumor size and the high cost of imaging. Emerging machine learning systems offer promise, with diagnostic precision ranging from 84.18% to 90.83%, supporting cancer identification and surgical decision-making [29,30]. Addressing misdiagnosis and delays in renal carcinoma detection remains a critical priority for health care providers.

Life expectancy is a primary concern in users’ inquiries about the 3 cancers. The standard query, “How long can one live with the diagnosis?” highlights that the most significant need for patients with cancer, in terms of treatment or intervention, is to minimize the negative impact of cancer on life expectancy. Currently, the growing population in Mainland China exacerbates the challenges posed by the morbidity and mortality of urologic cancers, affecting both the national health care system and personal lives [2]. As of 2019, the death rates for bladder cancer, kidney cancer, and prostate cancer in China were 50.39/1000, 40.09/1000, and 23.95/1000, respectively, with mortality rates gradually increasing with age [2]. Predictive models suggest that the morbidity and mortality rates for these 3 genitourinary cancers will continue to rise, although health care facilities are becoming more accessible in traditionally less-developed regions [31]. With improvements in perioperative management and increasing proficiency among surgeons in primary health care facilities, internet users have expressed concerns that could be addressed more promptly and validated by local health care professionals [31,32].

The search trend patterns across each cancer theme showed similarities, with an overall upward trend primarily driven by the topics of complaint and inquiry. The demand for cancer treatment and prognosis has generally declined, with the only exception being observed in prostate cancer treatment topics. Specifically, the search trends for bladder and kidney cancers increased until 2019, followed by a decline until a resurgence in 2021. For prostate cancer, turning points occurred in 2015 and 2020. The rising search trends may partly reflect real-world cancer incidence. Data from the GBD 2019 database indicated that the counts for bladder, kidney, and prostate cancers in 2019 were at least four times higher, with age-standardized incidence rates at least double those of 1990 [2]. This period also marked Baidu’s “golden age,” during which it became the primary source of information for internet users [13]. Although Baidu’s marketing strategies for channeling information have been criticized, they do not appear to have affected its dominant role in providing cancer-related information to users [13].

Despite the COVID-19 pandemic’s significant impact on regular medical activities and the shift in public attention toward outbreak management, online demand for information on tumor diseases, including genitourinary cancers, persisted and even increased in 2019 [6,33]. This demand was temporarily suppressed during the 2020-2021 pandemic peak but rebounded as government policies and health care responses evolved [34,35]. Search terms highlighted unresolved issues and user concerns about genitourinary cancers, emphasizing Baidu’s role as a trusted source of health-related information. These trends underscore the need to address user-identified health care gaps in future health policy optimization.

Geographically, searches for the 3 cancers were led by East China, accounting for approximately 30%, followed by North China at around 18%. Other regions showed similar levels of interest, each at approximately 10%, while the northwest region ranked last with about 8%.

Socioeconomic inequalities significantly influence health-seeking behavior and online health information searches [33,36]. In China, disparities in medical center density, health care costs, social security policies, and household finances shape patients’ decisions, with individuals from disadvantaged backgrounds often hesitating to seek treatment due to financial constraints [37]. Similarly, socioeconomic and educational factors affect online search behavior: higher-income individuals tend to exhibit greater digital literacy, use precise medical terminology, and seek reliable sources [38,39]. By contrast, lower-income groups face barriers such as limited internet access and lower trust in online information, often relying on symptom-based searches [40]. These differences underscore the impact of socioeconomic status on health awareness and access to accurate medical information [39,41].

Demographic data revealed similar interest in cancer topics across genders, though females expressed greater concern about kidney cancer prognosis. Bladder and kidney cancers have significantly higher incidence rates in males, at 5-fold and 2.5-fold, respectively. Interest in prostate cancer, despite being male-specific, indicates engagement extending beyond patients alone [2]. Agewise, searches for bladder and kidney cancers were common among users aged 20-39 years, with kidney cancer treatment queries peaking in the 40-49-year age group. Prostate cancer searches followed a similar pattern, except prognosis-related queries, which were predominantly from users aged 40-49 years. Bladder and kidney cancer incidence rates in individuals aged 20-39 years (25.48/100,000 and 1.51/100,000, respectively) were half of those observed in the 40-59-year age group, while prostate cancer incidence peaked in individuals over 60 years [2].

The disparity between incidence rates and concern levels across gender and age groups highlights the importance of health consciousness and awareness. The high cost, complex nursing requirements, and strict follow-up schedules associated with cancer treatment underscore that the disease burden affects not only individuals but also entire families [42]. Research indicates that women play a prominent leadership role within families, making approximately 80% of health care–related decisions [43-45]. The concerns and demands of women, as key decision makers within families, should not be underestimated. Although older adults are increasingly integrating internet technology into their daily routines, including health care–seeking activities, the persistent digital divide among older users remains a significant challenge [46]. Therefore, joint decision-making involving family members, rather than focusing solely on the patients’ needs, should be taken into account.

Our study found that only 15.74% of user search queries were categorized as relevant, reflecting a focus on trending topics, nonhealth issues, or vague terms, such as medication-related words. This low percentage suggests that public attention often shifts toward less scientifically grounded information, driven by societal trends, misinformation, or curiosity about lifestyle, diet, or alternative medicine [47,48]. The findings highlight the need for more effective dissemination of accurate cancer information and targeted educational campaigns to enhance public understanding of critical cancer issues. Furthermore, many users rely on general or indirect language in their searches, which may prevent their queries from being classified as relevant. This underscores the importance of promoting more precise health communication.

Overall, frequency, diagnosis, and symptom confirmation issues ranked among the top categories, accounting for 30.17% and 30.16%, respectively. Regarding popularity, interest in symptom confirmation ranked first, followed by diagnosis and health-related issues, each comprising approximately 15%. Similar patterns were observed in bladder and kidney cancers. However, in prostate cancer, while interest in diagnosis and symptom confirmation was dominant in terms of frequency, health care–related issues in prognosis garnered the most actual popularity.

In the topics of complaints and etiology inquiries, the most common concerns related to bladder and kidney cancers were hematuria and questions about its causes. As a marker of cancer risk, hematuria is often associated with underlying urologic malignancies. The standardized incidence ratio for overall urologic cancer risk peaks within the first 3 months following a hematuria diagnosis, reaching 14.15% [49]. Specifically, the standard incidence ratios for bladder, kidney, and prostate cancers at 3 months are 186.43%, 81.40%, and 14.18%, respectively [49]. Reports indicate that delayed responses to initial hematuria often lead to missed cancer diagnoses. Therefore, heightened awareness and timely investigation of its causes are recommended to prevent delays in cancer diagnosis [50,51].

We observed that several differential diagnoses were listed within the diagnosis topic. For bladder cancer, cystitis and prostate cancer were frequently mentioned. In the context of kidney cancer and prostate cancer, the most commonly referred differential diagnoses included renal cysts, renal kidney disease, prostatitis, and benign prostatic hyperplasia. These conditions must be ruled out before confirming a tumor diagnosis, suggesting the potential for users to engage in self-assisted cyber diagnosis [52].

Accurate evidence is essential for the correct diagnosis of cancer, as it helps identify tumor characteristics. In some cancer cases, even experienced experts may find judgment and decision-making complicated due to varying test results [53]. The risk of internet self-diagnosis in cancer warrants greater attention, as diagnosis delays and misleading treatments can have serious consequences [52]. For users with limited medical knowledge, it becomes even more challenging to identify accurate and useful information while maintaining reasonable and objective expectations [54]. Therefore, online health care information should be more instructive, neutral, and objective, enabling potential patients to better follow guidance from medical professionals [55].

### Limitations

Several limitations of this study should be acknowledged. First, although Baidu is the largest search engine in mainland China, its dominance is increasingly challenged by emerging search platforms and social media, which restricts the scope of online search behavior captured. Consequently, the daily BSI may not fully reflect the demands of all internet users. Second, regional differences in user preferences and political regulations result in the exclusion of searches by individuals legally accessing international platforms. Furthermore, as Baidu is a Chinese platform, searches conducted in foreign languages or by ethnic minorities may be systematically omitted, potentially introducing bias. Third, the absence of a real-time public cancer database is a significant limitation, as it prevents the integration of search data with clinical data for more accurate disease prediction and forecasting. These factors underscore biases in internet access and usage across different demographic groups, which should be considered when interpreting the results in the context of broader cancer awareness and digital health information–seeking behavior.

### Future Directions

This study is the first to address public concerns regarding the 3 major genitourinary cancers within the Chinese-speaking population. These cancers were selected due to their complexity and subtle onset, which often lead to delayed diagnoses and severe outcomes. By analyzing online search trends, this research provides valuable insights into patient perceptions and needs, offering a broader understanding of public demand. Future infodemiology studies should incorporate data from multiple search engines, social media platforms, and multilingual or minority groups to achieve a more comprehensive analysis of public health trends. Integrating such a system into a national cancer database could significantly enhance disease tracking, forecasting, and public health decision-making.

### Conclusions

Online searches and inquiries related to genitourinary cancers are on the rise. The depth of users’ information demands appears to be influenced by regional economic levels. Cancer treatment decision-making may often involve a family-centered approach. Insights from internet search data are potentially beneficial for medical professionals to better understand public interests and concerns, enabling them to provide more targeted and reliable health care services.

## Data Availability

The data sets generated and analyzed during this study are available from the corresponding author upon reasonable request.

## Appendix

Multimedia Appendix 1 List of keywords used in composite search index.

## Abbreviations

AAPC: average annual percentage change
APC: annual percentage change
BSI: Baidu Search Index
GBD: global burden of disease
TCM: traditional Chinese medicine
