# Supplementary material for: Analyzing Online Search Trends for Kidney, Prostate, and Bladder Cancers in China: Infodemiology Study Using Baidu Search Data (2011-2023)
Source: JMIR Cancer. 2025 Mar 14;11:e57414. doi: 10.2196/57414 (PMC11953601; doi:10.2196/57414)
Supplement: Multimedia Appendix 1 [file cancer_v11i1e57414_app1.docx]

| List of keywords used in composite search index | | |  |
| --- | --- | --- | --- |
| Domain of terms | Available term in Search engine | English equivilent terms | |
| Complaint | 膀胱癌 | Bladder cancer | |
|  | 膀胱肿瘤 | Bladder tumor | |
|  | 肾癌 | Kidney cancer | |
|  | 肾肿瘤 | Kidney tumor | |
|  | 肾脏肿瘤 | Kidney tumor | |
|  | 肾部肿瘤 | Tumor in Kidney | |
|  | 左肾肿瘤 | Left kidney tumor | |
|  | 恶性肾肿瘤 | Malignant kidney tumor | |
|  | 前列腺癌 | Prostate Cancer | |
| Inquiry | 膀胱癌早期是什么症状 | What are the early symptoms of bladder cancer | |
|  | 膀胱癌的早期症状 | Early symptoms of bladder caner | |
|  | 膀胱癌症状 | Bladder cancer symptoms | |
|  | 膀胱癌分期 | Staging of bladder cancer | |
|  | 膀胱癌有什么症状 | What are the symptoms of bladder cancer | |
|  | 膀胱癌的症状 | Bladder cancer symptoms | |
|  | 膀胱癌晚期 | Late stage bladder cancer | |
|  | 膀胱癌转移 | Bladder cancer metastasis | |
|  | 膀胱癌晚期症状 | Bladder cancer late-stage symptoms | |
|  | 肾癌早期的五个表现 | 5 Signs of early kidney cancer | |
|  | 肾癌的早期症状 | Early symptoms of kidney cancer | |
|  | 肾癌晚期 | Late-stage kidney cancer | |
|  | 肾癌症状 | Kidney cancer symptoms | |
|  | 肾癌分期 | Kidney cancer staging | |
|  | 肾癌转移 | Kidney cancer metastasis | |
|  | 肾肿瘤的早期症状 | Early symptoms of kidney cancer | |
|  | 前列腺癌的早期症状 | Early symptoms of prostate cancer | |
|  | 前列腺癌症状有哪些 | What are the symptoms of prostate cancer | |
|  | 前列腺癌症状 | Symptoms of prostate cancer | |
|  | 前列腺癌的症状 | The Symptoms of prostate cancer | |
|  | 前列腺癌分期 | Prostate cancer staging | |
|  | 前列腺癌晚期 | Late-stage prostate cancer | |
|  | 前列腺癌骨转移 | Bone metastasis of prostate cancer | |
| Treatment | 膀胱癌治疗 | Treatment of bladder cancer | |
|  | 肾癌的治疗 | Treatment of kidney cancer | |
|  | 前列腺癌治疗 | Treatment of prostate cancer | |
|  | 前列腺癌的治疗 | The treatment of prostate cancer | |
| Prognosis | 膀胱癌能活多久 | How long one can live with diagnosed Bladder cancer | |
|  | 肾癌晚期能活多久 | How long one can live with diagnosed Bladder cancer | |
|  | 前列腺癌能活多久 | How long one can live with diagnosed Bladder cancer | |
|  | | |  |
